# Stored Autogenous Particulate Dentin for Alveolar Ridge Preservation: One‐Year Clinical and Histological Case Study

**DOI:** 10.1002/ccr3.72606

**Published:** 2026-04-26

**Authors:** Saif Mohi Eddin, Obada Mandil, Dimitris Tatakis, Hani ElNahass

**Affiliations:** ^1^ Department of Periodontics, School of Dentistry Cairo University Giza Egypt; ^2^ Department of Periodontics, School of Dental Medicine Case Western Reserve University Cleveland Ohio USA; ^3^ Department of Periodontics and Oral Medicine, School of Dentistry University of Michigan Ann Arbor Michigan USA

**Keywords:** alveolar ridge augmentation, cone‐beam computed tomography, dentin, histology, transplantation‐ autologous

## Abstract

This case report describes the clinical, radiographic, and histological outcomes of alveolar ridge preservation using chairside‐prepared autogenous dentin graft stored at room temperature. A 25‐year‐old female underwent extraction of a non‐restorable maxillary premolar followed by ridge preservation using autogenous dentin graft prepared and stored 3 weeks earlier. Healing was uneventful. One‐year radiographic and histological evaluation demonstrated adequate bone formation with newly formed vital bone and no evidence of inflammation or residual dentin particles. This case contributes to emerging evidence supporting stored autogenous dentin as a potential grafting material.

## Key Clinical Message

Stored autogenous dentin particulates can be successfully used for alveolar ridge preservation. This case demonstrates a simple chairside protocol for dentin preparation and storage at room temperature, with favorable clinical and histological outcomes observed at a 1‐year follow‐up, supporting its viability as a bone grafting material.

## 1 Introduction

Following tooth extraction, alveolar ridge resorption can be observed throughout life, with the highest rate in the first 3–6 months [1]. The reported ranges of bone loss are 29%–63% horizontally and 11%–22% vertically after 6 months [1]. Others reported approximately 50% of horizontal bone loss occurring within the first 12 months following the extraction of premolar or molar teeth [2]. However, the extent of post‐extraction resorption has been shown to vary by anatomical location [3], and recent findings indicate that maxillary premolar sites can exhibit approximately 34.3% vertical and 55.7% horizontal bone loss within 6 months [4]. This loss of alveolar bone often results in inadequate ridge dimensions for implant placement [5].

To overcome this obstacle, the alveolar ridge preservation (ARP) procedure was developed [6]. Compared to non‐preserved sites, extraction sites treated with ARP experience reduced bone loss [7, 8]. Various biomaterials, including xenografts, allografts, and synthetic substitutes, have been investigated for ARP [7, 8]. However, freshly prepared non‐demineralized autogenous dentin graft for ARP was introduced in 2014 [9]. This biomaterial has demonstrated efficacy as an alternative bone substitute for alveolar ridge preservation, sinus lift procedures, and the treatment of class II furcation defects [9, 10, 11].

Despite its potential benefits, the use of freshly prepared dentin is not without limitations, including the finite quantity of available material and the inevitable prolongation of the time needed to complete the clinical procedure in order to prepare the graft. These limitations could be overcome by using stored dentin graft material, which may offer comparable clinical benefits to fresh dentin, as growth factors within human teeth remain intact for thousands of years [12].

Although a few studies have discussed the potential for storing non‐demineralized dentin, there remains a lack of clinical investigations specifically evaluating the bone healing response achieved with such stored graft materials. Hallel et al. utilized chairside‐prepared fresh dentin for grafting procedures and briefly mentioned that any excess dentin could be stored at room temperature; however, no clinical evaluation of the outcomes associated with stored dentin was conducted [9]. In a different approach, Kim et al. reported the storage of extracted teeth in a refrigerator prior to graft preparation. Additionally, they indicated that tooth‐derived graft materials could be stored at room temperature after undergoing a series of processing steps, including dehydration, defatting, lyophilization, and sterilization with ethylene oxide [13]. Nevertheless, robust clinical evidence is still lacking to define or validate an appropriate protocol for chairside dentin storage and to assess its associated clinical outcomes.

Hence, this report aims to describe a case study where the use of stored autogenous dentin as particulate grafting material for ARP procedure was evaluated both clinically and histologically.

## 2 Materials and Methods

### 2.1 Case History/Examination

A 25‐year‐old female was referred to the Department of Periodontology at Cairo University for tooth extraction and ARP in anticipation of future implant placement. The patient was medically fit. Comprehensive examination revealed localized Stage II Grade B periodontitis, according to the 2017 World Workshop classification system [14], and non‐restorable teeth #15, #24, and #25 due to carious lesions. The patient accepted a treatment plan including periodontal phase I therapy, tooth extraction, ARP, and subsequent implant placement and provided written informed consent.

### 2.2 Clinical Procedures

#### 2.2.1 Initial Treatment Phase

Oral hygiene instructions and scaling and root planing were accomplished before the tooth extraction. Extraction of the maxillary left premolar teeth was performed under standard clinical protocol, which included preoperative 0.12% chlorhexidine rinse (Macro Group Pharmaceutical, Egypt) for one minute, profound local infiltration anesthesia (2% mepivacaine HCl and levonordefrin, 1:20,000 Alexandria Co. for Pharmaceuticals, Egypt), minimally traumatic extraction using flexible periotomes and forceps, and curettage of the sockets. The fresh autogenous dentin graft was then prepared (see below) and used for ARP at the two edentulous sites. The remaining dentin particles were stored to be used during the performance of ARP following the extraction of the maxillary right second premolar (#15) at a subsequent appointment.

#### 2.2.2 Chairside Autogenous Dentin Graft Preparation

**FIGURE 1:**
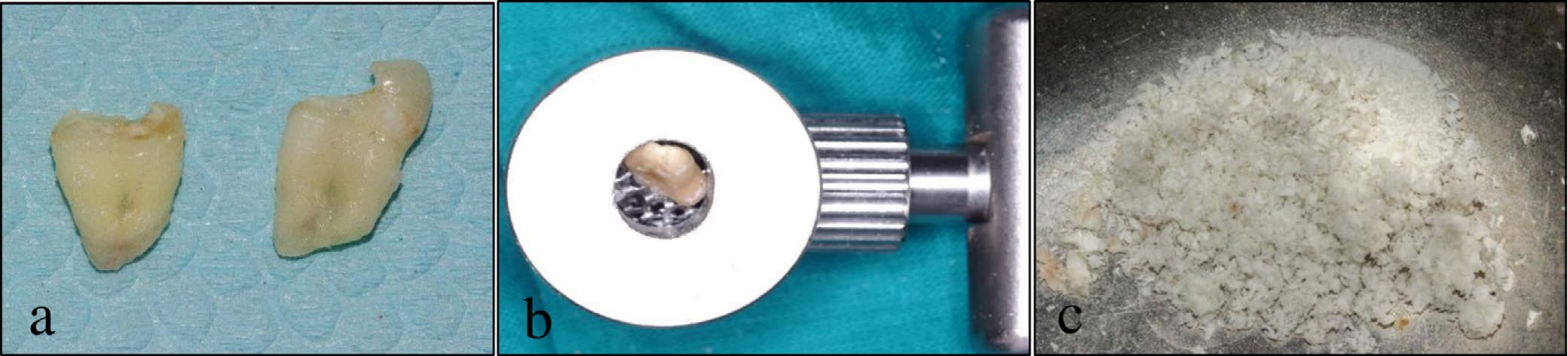
(a) Teeth number #12 and #13 after cleaning before enamel removal; (b) manual bone mill for teeth grinding; (c) dentin particulates in a sterile dish.

The dentin graft was prepared following the protocol described by Elfana et al. [15], except that the enamel was removed during the preparation process. Extra‐orally, a carbide bur was used to remove enamel, any carious tooth structure, remnants of the periodontal ligament, cementum, and calculus from teeth #24 and #25, under copious saline irrigation. Root canals were mechanically cleaned using manual K files ranging in size (10–35) (Figure 1a). Subsequently, the prepared dentin was dried and ground using a manual bone mill (GMD‐02 Gold Mill, MCT Bio, Korea) (Figure 1b).

The freshly prepared particulate dentin graft was disinfected by immersion in basic ethanol solution (Chemajet Chemical and Pharmaceutical Industries, Egypt) for 10 min, then rinsed twice using sterile saline solution and dried with sterile gauze. It was then used for ARP on the maxillary left side. The remaining dentin graft material was stored in a sterile container at room temperature until the next scheduled ARP procedure (Figure 1c).

#### 2.2.3 ARP Procedure Using Stored Particulate Dentin Graft

**FIGURE 2:**
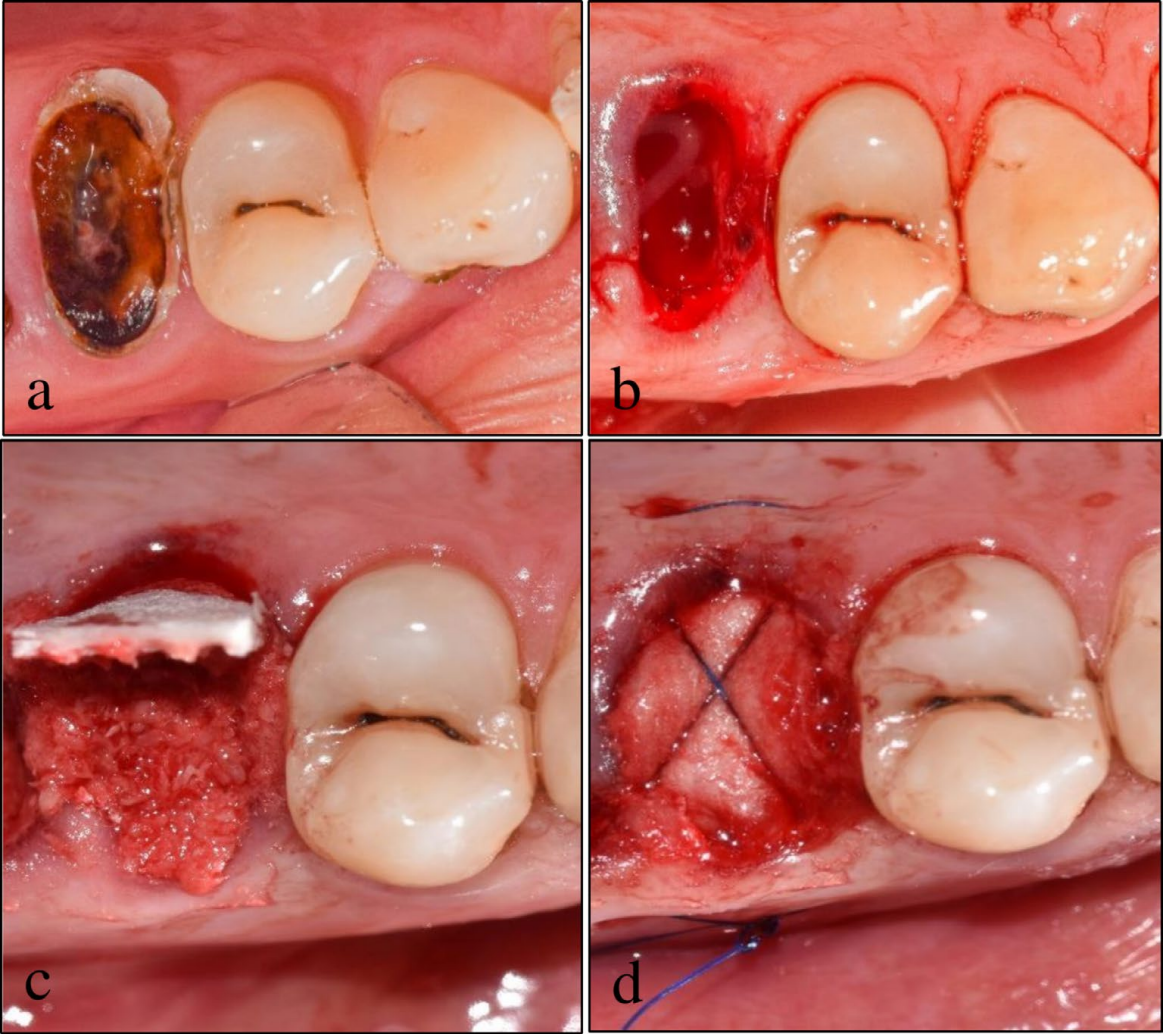
(a) Preoperative clinical presentation of tooth #4; (b) post‐extraction of tooth #4 (c) stored dentin particulates properly packed in the socket; (d) collagen barrier membrane covering the socket and secured by X suture technique.

3 weeks after graft preparation, extraction of tooth #15, following the same standard clinic protocol detailed above, and ARP were performed using the remaining stored portion of the graft prepared from teeth #24 and #25 (Figure 2a,b). For ARP, the stored graft was lightly hydrated with sterile saline and placed in the socket (Figure 2c). A flapless socket closure was accomplished using a collagen resorbable membrane (Hypro‐ Sorb, Bioimplon GmbH, Germany) and stabilized by a 5‐0 polypropylene suture [16] (Assut Medical Sarl, Switzerland) (Figure 2d).

#### 2.2.4 Postoperative Protocol

The patient was given instructions on oral hygiene and postoperative medications, which included 0.12% chlorhexidine mouthwash BID, amoxicillin 500 mg TID, and ibuprofen 400 mg every 8 h for a week. She was scheduled for follow‐up at 2 weeks postoperatively.

#### 2.2.5 Bone Core Harvest

Twelve months after the ARP procedure, at the time of implant placement, a 2‐mm internal diameter trephine drill was utilized to harvest a 6‐mm long core biopsy from the center of the preserved site. Sequential drilling followed according to the manufacturer's instructions, and a 3.75 × 11.5 mm dental implant (NEODENT GRAND MORSE IMPLANT SYSTEM) was placed, achieving 30 N/cm^2^ insertion torque.

### 2.3 Radiographic Analysis

**FIGURE 3:**
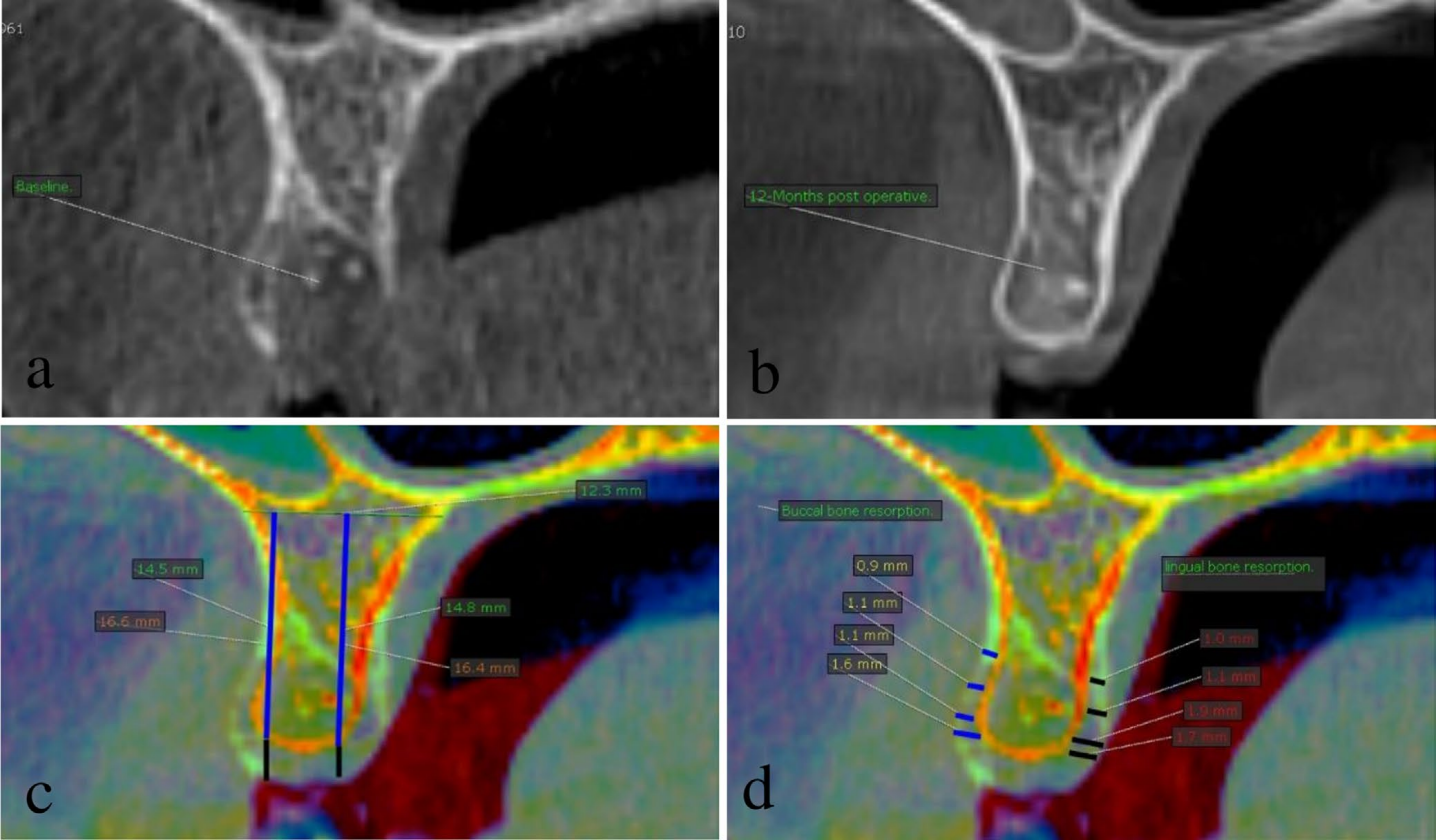
(a) Baseline CBCT scan showing alveolar ridge configuration immediately after the ARP procedure; (b) 1‐year follow‐up CBCT scan demonstrating bone formation at the site of preservation with proper ridge morphology; (c) a coronal view of the superimposed baseline (the black vertical line) and follow‐up (the blue vertical line) scans to demonstrate the vertical dimensional change that has occurred throughout a year of follow‐up; (d) a coronal view of the superimposed baseline and follow‐up scans to demonstrate the buccolingual dimensional change that has occurred throughout a year of follow‐up.

Baseline and 12‐month Cone Beam Computed Tomography (CBCT) scans were performed (Figure 3a,b) and superimposed using specialized CBCT analysis software (OnDemand3DApp‐ Fusion), to measure the amount of vertical and horizontal bone changes. All measurements were referenced to the existing crestal bone at each time point, taking into consideration the vertical remodeling over the follow‐up period.

Vertical bone changes were measured on the superimposed coronal view. A horizontal reference line was drawn at the lowest point of the sinus floor. Perpendicular lines to the horizontal reference line were extended to the highest crestal points on both the buccal and lingual aspects of the ridge (Figure 3c), as well as to the mid‐crestal point, marking the height of the alveolar ridge at baseline and 12‐month postoperatively.

Horizontal bone changes were measured at four different horizontal levels on the superimposed coronal view, at 1, 2, 4, and 6 mm apically to the crest of the alveolar ridge at follow‐up. All of the horizontal measurement lines were perpendicular to the vertical reference line, which was parallel to the long axis of the ridge (Figure 3d).

### 2.4 Histological Analysis

The bone core specimen, obtained at the time of implant placement, was placed into 10% neutral‐buffered formalin, decalcified by EDTA, and embedded in paraffin wax. Longitudinal 5‐μm thick sections were prepared and stained with hematoxylin and eosin (H&E) for routine light microscopy. H&E‐stained sections at 40× and 100× magnification were used for qualitative analysis and description of the tissue response to the dentin graft after 12 months, specifically documenting the presence of residual dentin particles and any associated inflammatory reaction. The histological evaluation was performed by a single experienced oral pathologist.

## 3 Results

### 3.1 Clinical Examination

At the 2‐week postoperative visit, the patient reported no complaints and tissue healing was uneventful. At the same visit, sutures were removed, and postoperative instructions were reiterated. Subsequently, the patient was unable to attend the scheduled 4‐month follow‐up appointment due to compelling personal circumstances. 12 months after the ARP procedure, she presented for implant placement. Clinical examination revealed mature soft tissue over the grafted site. The alveolar ridge present at 12 months appeared adequate for placement of a dental implant, without the need for any additional bone augmentation at that time. The patient was satisfied with her experience with the ARP procedure and the fact that no further bone grafting would be needed prior to implant placement.

### 3.2 Radiographic Examination

The radiographic examination confirmed the clinical impression that adequate bone was available for dental implant placement, without the need for any additional bone augmentation at that time. The recorded vertical and horizontal bone changes are reported in detail in Tables 1 and 2, respectively.

**TABLE 1:** Vertical bone changes of the alveolar ridge at baseline and 12 months after the surgery (Figure 3c).

	Baseline	Follow‐up	Changes after 1‐year
Ridge height buccal	16.6 mm	14.5 mm	−2.1 mm (12.6%)
Ridge height palatal	16.4 mm	14.8 mm	−1.6 mm (9.7%)
Ridge height at mid‐crestal	17.7 mm	15.7 mm	−2 mm (11.3%)

**TABLE 2:** Horizontal bone changes of the alveolar ridge at baseline and 12 months after the surgery (Figure 3d).

Ridge width at	Baseline	Follow‐up	Changes After 1‐Year	Detailed changes buccal palatal	
1 mm apical to the crest	9.1 mm	5.8 mm	3.3 mm (36.2%)	1.6 mm	1.7 mm
2 mm apical to the crest	9.5 mm	6.5 mm	3 mm (31.5%)	1.1 mm	1.9 mm
4 mm apical to the crest	8.9 mm	6.7 mm	2.2 mm (24.7%)	1.1 mm	1.1 mm
6 mm apical to the crest	8 mm	6.1 mm	1.9 mm (23.7%)	0.9 mm	1.0 mm

### 3.3 Histologic Findings

**FIGURE 4:**
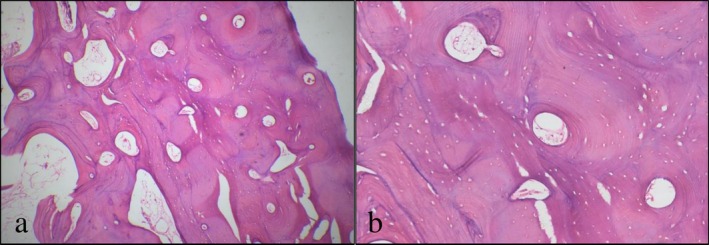
(a) H&E‐stained section of the core biopsy at 40× magnification, showing bone formation with no remnants of dentin particles; (b) the same view at 100× magnification, revealing finer details of the formed bone, with no signs of inflammation.

The stained section of the core biopsy revealed normal bone trabeculae with reversal lines, viable osteocytes, and well‐vascularized bone marrow. There was no evidence of residual graft material or signs of inflammation (Figure 4a,b).

## 4 Discussion

This report aimed to document the use of stored autogenous particulate dentin graft for ARP. Radiographically, buccolingual bone loss measured 2 mm apical to the bone crest was 31.5%, and vertical bone loss at the mid‐crest was 11.3%. These results demonstrate substantially less bone loss compared to the natural healing of non‐grafted maxillary premolar sockets (55.7% horizontal and 34.3% vertical) bone loss reported within 6 months [4]. Consequently, in this case, the alveolar ridge was preserved using stored autogenous dentin and no additional bone grafting was required for implant placement.

Histological analysis of the harvested bone core biopsy revealed that the graft particles were completely resorbed and replaced by vital bone. In comparison, a clinical study utilizing fresh dentin, showed 63% new bone formation after 7 months of healing, with signs of ongoing remodeling around residual dentin particles [17].

Evidence supporting the clinical application of stored autogenous dentin as a bone substitute throughout the literature is scarce. However, fresh autogenous dentin graft has been demonstrated to be efficient for ARP [10] and in a comparison with xenograft, fresh dentin graft showed more newly formed vital bone and less residual graft particles [18]. Similarly, another study demonstrated that when fresh dentin was compared to beta‐tricalcium phosphate (β‐TCP) alloplast and allograft, the dentin grafts exhibited less bone loss and a higher proportion of mature bone formation compared to allograft and alloplast groups [19].

The process of autogenous dentin storage can be predictable and does not require special equipment, as dentin particles represent cell‐free mineralized matrix, and their autogenous origin practically eliminates the likelihood of any cross‐reaction. In the 1970s, foundational studies conducted by Urist et al. and Register et al. showed that dentin possesses a significant amount of growth factors, such as bone morphogenetic proteins (BMPs) and transforming growth factor beta (TGF‐β), which enhance its osteoinductivity and promote new bone formation [20, 21]. Moreover, growth factors such as BMP‐2, insulin growth factor II (IGF‐II), and TGF‐β remain viable and efficient for a prolonged period within the dentin matrix; thus, the osteoinductivity of the dentin particles is preserved [12].

Furthermore, dentin particles could exhibit good osteoconductive properties due to the porous structure of the dentinal tubules which facilitates osteocyte attachment to the particles [22]. The mineral content of dentin could compensate for the high resorption rate accompanied by the autogenous bone graft [23, 24]. Additionally, the structural and compositional similarities between dentin and alveolar bone may contribute to a favorable biological response to the dentin grafts by the human body [25]. Additionally, a clinical study has reported that tooth roots could be an alternative for autogenous bone blocks in lateral ridge augmentation procedures [26].

Autogenous dentin grafting is inherently limited to patients who are having a suitable tooth extracted, a fact that may restrict its clinical applicability. However, studies have shown that non‐demineralized and demineralized autogenous tooth grafts can be stored at room temperature for a long time [9, 13, 27]. Dentin storage could be useful in several situations, given that excess graft material could result from a procedure, since the volume of dentin particles produced by tooth grinding is twice that of the original tooth [9]; this would be especially helpful in cases of extraction of multiple teeth. Moreover, teeth extracted during third molar or deciduous tooth extraction, or for orthodontic purposes, can be stored for future use as graft material, offering an additional resource for later procedures. Thereby, if storage is a choice, 1000s of extracted teeth could be saved as a non‐synthetic bone graft substitute.

A case study, by its nature, has limitations. This case study describes a single patient, which limits the generalizability of the findings. Although most post‐extraction ridge dimensional changes occur within the first 3–6 months, the present evaluation was limited to a single 12‐month time point; nevertheless, this follow‐up remains clinically relevant and comparable to prior ridge preservation studies. Additionally, the absence of a direct comparison between stored and fresh dentin restricts conclusions regarding their relative efficacy. The graft particles in this case were stored for only 3 weeks, and further research is needed to optimize storage protocols. Moreover, the employed disinfection approach requires further investigation, as the use of a basic ethanol solution does not guarantee complete sterilization of the biomaterial; it reduces microbial load while preserving the biological activity of dentin‐derived proteins. Future research should assess the clinical applicability of stored dentin grafts in larger, controlled studies.

## 5 Conclusion

This case study demonstrates that stored autogenous particulate dentin could be a viable grafting material for alveolar ridge preservation. Histological analysis confirmed its complete resorption and replacement with new bone. Stored dentin could be an alternative biomaterial to complement the available bone substitutes. Further studies are needed to optimize storage protocols and confirm its broader clinical applications.

## Author Contributions

**Saif Mohi Eddin:** conceptualization, data curation, investigation, writing – original draft, writing – review and editing. **Obada Mandil:** data curation, writing – original draft, writing – review and editing. **Dimitris Tatakis:** data curation, writing – review and editing. **Hani ElNahass:** supervision, writing – review and editing.

## Funding

The authors have nothing to report.

## Consent

The patient provided written consent for treatment and for publication of this report.

## Conflicts of Interest

The authors declare no conflicts of interest.

## Data Availability

All data regarding this article will be provided upon request from the authors. The corresponding author can provide the data that were utilized to support the study's conclusions upon request.
